# Population Pharmacokinetics of Antimalarial Naphthoquine in Combination with Artemisinin in Tanzanian Children and Adults: Dose Optimization

**DOI:** 10.1128/aac.01696-21

**Published:** 2022-04-25

**Authors:** Ali Mohamed Ali, Kamunkhwala Gausi, Said A. Jongo, Kamaka R. Kassim, Catherine Mkindi, Beatus Simon, Ali T. Mtoro, Omar A. Juma, Omar N. Lweno, Conrad H. Gwandu, Bakari M. Bakari, Thabiti A. Mbaga, Florence A. Milando, Ali Hamad, Seif A. Shekalaghe, Salim Abdulla, Paolo Denti, Melissa A. Penny

**Affiliations:** a Swiss Tropical and Public Health Institute, Basel, Switzerland; b University of Basel, Basel, Switzerland; c Ifakara Health Institute, Bagamoyo, Tanzania; d Division of Clinical Pharmacology, Department of Medicine, University of Cape Town, Cape Town, South Africa

**Keywords:** dose optimization, malaria, population pharmacokinetics

## Abstract

The combination antimalarial therapy of artemisinin-naphthoquine (ART-NQ) was developed as a single-dose therapy, aiming to improve adherence relative to the multiday schedules of other artemisinin combination therapies. The pharmacokinetics of ART-NQ has not been well characterized, especially in children. A pharmacokinetic study was conducted in adults and children over 5 years of age (6 to 10, 11 to 17, and ≥18 years of age) with uncomplicated malaria in Tanzania. The median weights for the three age groups were 20, 37.5, and 55 kg, respectively. Twenty-nine patients received single doses of 20 mg/kg of body weight for artemisinin and 8 mg/kg for naphthoquine, and plasma drug concentrations were assessed at 13 time points over 42 days from treatment. We used nonlinear mixed-effects modeling to interpret the data, and allometric scaling was employed to adjust for the effect of body size. The pharmacokinetics of artemisinin was best described by one-compartment model and that of naphthoquine by a two-compartment disposition model. Clearance values for a typical patient (55-kg body weight and 44.3-kg fat-free mass) were estimated as 66.7 L/h (95% confidence interval [CI], 57.3 to 78.5 L/h) for artemisinin and 44.2 L/h (95% CI, 37.9 to 50.6 L/h) for naphthoquine. Nevertheless, we show via simulation that patients weighing ≥70 kg achieve on average a 30% lower day 7 concentration compared to a 48-kg reference patient at the doses tested, suggesting dose increases may be warranted to ensure adequate exposure. (This study has been registered at ClinicalTrials.gov under identifier NCT01930331.).

## INTRODUCTION

There is a pressing need to develop novel antimalarials and to assess existing antimalarials to treat malaria. New therapies are needed to address efficacy declines of several approved artemisinin-based combination therapies (1) and the threat of emerging drug resistance. Artemisinin resistance for Plasmodium falciparum is present on the Thai-Cambodia border (2–5) and now present in Africa (6). Current artemisinin-based combination therapy (ACT) treatment courses are generally based on a recommended 3-day regimen to ensure sufficient artemisinin exposure. However, exposure might be compromised by poor patient adherence (7–10) and is thus thought to be a factor in the development of drug resistance (11). Single-dose therapies have been explored to improve patient adherence and are preferable to a 3-day regimen. An oral single-dose regimen was developed for the combination of artemisinin and naphthoquine phosphate (ART-NQ), a new-candidate artemisinin-based combination therapy (12). However, despite the potential that a single dose may improve adherence, cure rates with any new ACT, including ART-NQ, should be ensured or improved with multiday dosing (13, 14), and there is thus a need to assess both the efficacy and pharmacokinetics (PK) of different regimens of ART-NQ or new ACTs. Unfortunately, naphthoquine was recently shown to be potentially associated with induced central nervous system toxicity in animal studies as well as hepatic vasculocentric toxicity (15). Here, we report for completeness previous pharmacometrics and model-based assessment of naphthoquine completed prior to new toxicity studies.

Artemisinin has been relatively well studied; however, naphthoquine alone or in combination with artemisinin has been less studied. Artemisinin has a fast-acting parasiticidal action (16, 17), but because of its very short terminal half-life, when used alone it has the disadvantage of high recrudescence rates (18) and risk of drug resistance (19, 20). Naphthoquine, on the other hand, has a longer terminal half-life (11, 21) and larger oral bioavailability (96.4%). Artemisinin is not completely absorbed when taken orally; its relative bioavailability (F) was reported to be 32% (22). Human liver microsome studies report that it is metabolized primarily by CYP2B6, with a probable secondary contribution of CYP3A4 and CYP2A6 (23) into 4 inactive metabolites (24). The degree of binding for artemisinin to human serum or plasma proteins was reported to be 64% (25). Artemisinin is cleared almost entirely by the liver, and the total amount of unchanged artemisinin excreted in urine is less than 1% of the dose (26). Naphthoquine is metabolized in liver and excreted from urine (21); however, its metabolism and protein binding are still unknown. It has higher cure rate than artesunate (27), but a slower onset of parasite killing (28) compared to artemisinin and its derivatives. Combining these two drugs may have the advantage of overcoming their individual weaknesses and hence reduce the pressure of drug resistance. Artemisinin reduces the parasite number very rapidly, and the residual parasites are then exposed to relatively high levels of the partner drug (29), which remains in the bloodstream for longer.

The current manufacturer’s recommended dosage of ART-NQ for a 50-kg individual is a single dose containing 1,000 mg artemisinin and 400 mg naphthoquine. The dose regimen for young children is then scaled down using body weight targeting the same mg/kg (28). As previously reported for several drugs (30–35), there is some concern that dosing recommendations may not be optimal for some subgroups of patients, including young and/or malnourished children. For malaria, this may result in lower exposure and malaria recrudescence: for example, in a study conducted in Burkina Faso, young children (2 to 5 years) received lower exposure than older children (6 to 10 years) after receiving piperaquine doses based on allometric scaling (36).

ART-NQ is registered by Kunming Pharmaceuticals (Kunming, China) and has been used for treatment of uncomplicated malaria for patients of all ages including young children. The safety and efficacy of ART-NQ have been assessed in several clinical studies (28); however, no study has been conducted to assess exposure in an African population. In 2014, a study was undertaken to confirm cardiac safety profile, tolerability, and efficacy of ART-NQ in a Tanzanian setting, as well as its pharmacokinetic properties. The objective of the present analysis is to develop a population pharmacokinetic model of naphthoquine in a Tanzanian population and thus assess if the current dosing in children results in similar exposure levels to adults. Furthermore, the resulting model will be used to explore alternative optimal dosage regimens via simulation. Since the study reported here, naphthoquine was found to be associated with a potential for toxic side effects (15); however, its role in antimalaria treatment is unclear. Nevertheless, we report results for future pharmacokinetic and exposure studies.

## RESULTS

A total of 29 Tanzanian patients with uncomplicated falciparum malaria were enrolled. The median age (range, interquartile range [IQR]) and weight at baseline were 13.1 (6.0 to 56.0, 8.1 to 21.1) years and 32.0 (20 to 84, 22.0 to 54.1) kg, respectively. Baseline characteristics of the study population are shown in Table 1. Overall, the median artemisinin dose was 18.5 (IQR, 16.7 to 18.8) mg/kg, and that of naphthoquine was 7.4 (IQR, 6.7 to 7.5) mg/kg.

**TABLE 1:** Baseline characteristics of the study population^*a*^

Baseline characteristic	Result for patients			
	6–10 yr	11–17 yr	≥18 yr	All
Total no. of patients	12	6	11	29
No. of males/females	3/9	4/2	8/3	15/14
Median age, yr (IQR)	7.1 (6.7–9.0)	13.5 (12.9–14.1)	26.6 (21.0–44.9)	13.1 (8.1–21.1)

Enrollment demographics and vital and laboratory parameters				
Median wt, kg (IQR)	20.0 (20.0–24.5)	37.5 (26.0–48.0)	55.0 (51.0–64.0)	32.0 (22.0–54.1)
Median ht, cm (IQR)	120.5 (117.0–126.5)	149.0 (136.0–162.0)	162.0 (155.0–172.0)	145.0 (122.0–162.0)
Median body mass index, kg/m^2^ (IQR)	14.7 (14.1–15.5)	15.8 (14.1–20.5)	20.4 (19.0–25.3)	16.4 (14.6–20.4)
GM parasitemia, parasites/μL (95 % CI)	951.9 (430.8–2103.4)	697.6 (236.5–2057.6)	417.0 (227.3–764.8)	652.7 (427.8–995.8)
Median hemoglobin, g/dL (IQR)	11.8 (11.2–12.3)	12.0 (11.1–12.7)	13.7 (11.8–14.3)	12.2 (11.3–13.7)
Median white blood cell count, 10^3^/μL (IQR)	9.1 (8.1–10.5)	5.3 (4.9–7.3)	4.9 (4.2–5.1)	6.1 (4.9–8.6)
Median red blood cell count, 10^3^/μL (IQR)	4.6 (4.4–4.9)	4.8 (4.5–5.1)	4.9 (4.4–5.4)	4.8 (4.5–5.0)
Median hematocrit, % (IQR)	35.5 (33.5–37.2)	36.0 (32.4–37.7)	39.9 (34.6–41.8)	36.4 (33.5–39.8)

Dosing information				
ART median total dose, mg/kg (IQR)	18.8 (18.6–18.8)	18.7 (17.4–20.8)	16.9 (15.4–18.5)	18.5 (16.7–18.8)
NQ median total dose, mg/kg (IQR)	7.5 (7.4–7.5)	7.5 (7.0–8.3)	6.8 (6.2–7.4)	7.4 (6.7–7.5)

a A percentage can be more or less than 100% due to a rounding error. ART, artemisinin; NQ, naphthoquine; GM, geometric mean.

### Pharmacokinetic modeling.

**FIG 1:**
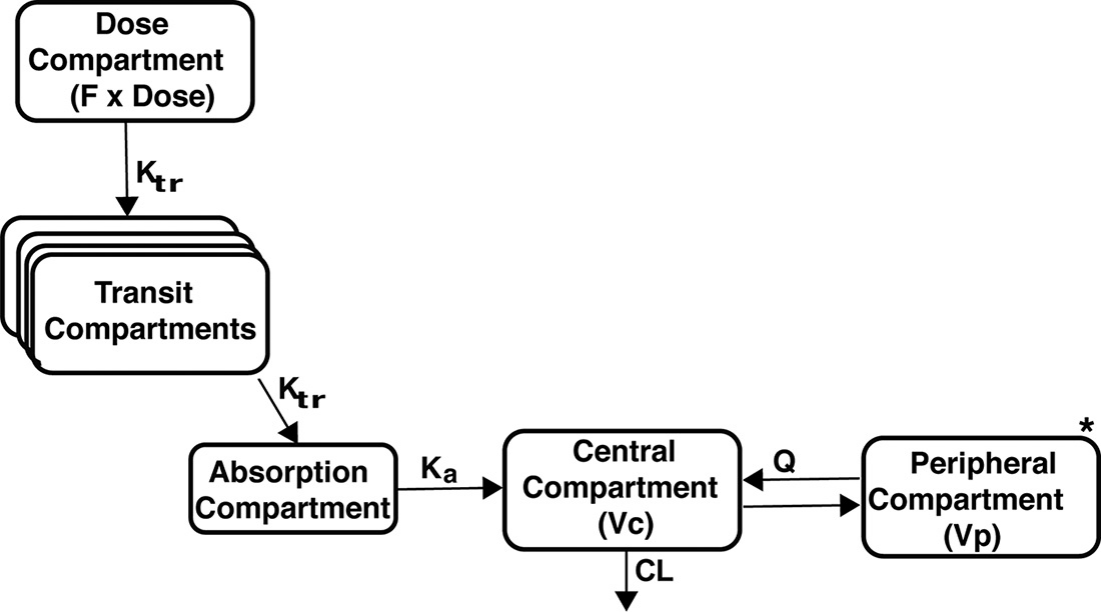
Structural presentation of the final model describing population pharmacokinetics for artemisinin and naphthoquine in Tanzanian malaria patients. F, oral bioavailability; K_tr_, first-order transit rate constant; K_a_, absorption rate constant; CL, clearance; Vc, central volume of distribution; Q, intercompartmental clearances; Vp, peripheral volumes of distribution. *, peripheral compartments apply only to naphthoquine.

**FIG 2:**
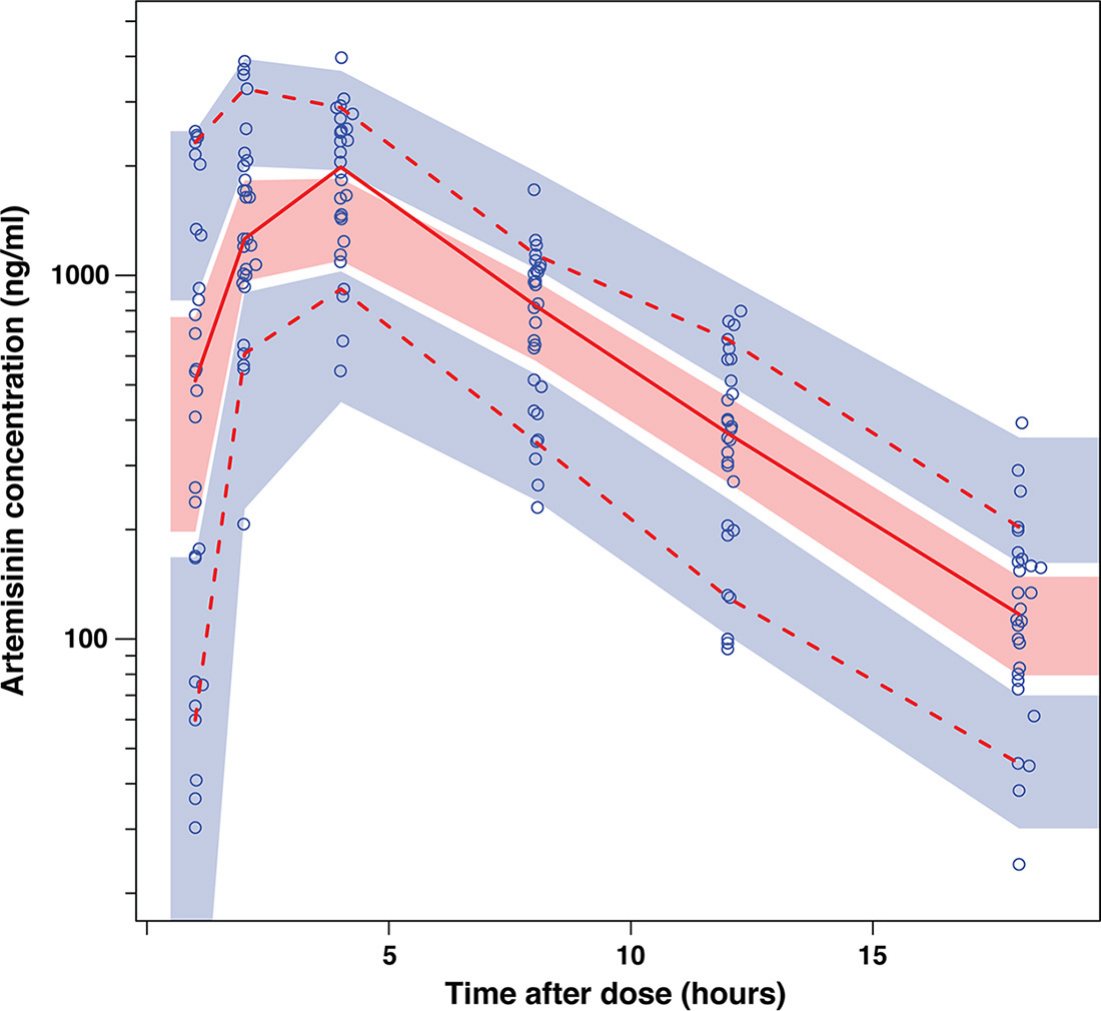
Visual predictive check of the final model describing the plasma concentration of artemisinin versus time in uncomplicated malaria patients from Tanzania. Open circles are the observed data points, solid and dashed lines are the 50th, 5th, and 95th percentiles of the observed data, and shaded areas are the simulated (*n* = 1,000) 95% confidence intervals for the same percentiles.

For artemisinin, 6 samples after the dose administration were collected in each of the 29 patients as per protocol, and none of the 174 samples was below the limit of quantification. One subject who was a slower absorber for artemisinin was not included in the PK of artemisinin because that would influence the analysis. Twenty-nine assays were excluded from the analysis after confirming that the concentration before the dose were below the limit of quantification, as expected. The observed artemisinin concentration-time data were best described by a one-compartment disposition model with transit compartment absorption. The transit compartment model was superior to a lag time model (change in objective function value [ΔOFV] = −59.5 versus −21.3). The final structural model is shown in Fig. 1, with parameter estimates given in Table 2. Incorporating body weight as an allometric function on clearance and volume parameters resulted in a better fit than the base model for artemisinin (ΔOFV = −17.9 and −4.6, respectively), and also interindividual variability decreased for clearance and volume by 16% and 3.3%, respectively. Testing fat-free mass as an alternative body size descriptor did not improve the fit. No other available covariate was significant. For a typical adult patient weighing 55 kg, the value of clearance was 66.7 L/h. A visual predictive check for the final model (*n* = 1,000) is depicted in Fig. 2, and basic goodness-of-fit diagnostic plots are presented in Fig. S1A in the supplemental material: these plots showed no overall obvious model misspecification.

**TABLE 2:** Parameter estimates of the population pharmacokinetic model for artemisinin and naphthoquine

Parameter^*a*^	Estimate (95% CI) for^*b*^:	
	Artemisinin	Naphthoquine
CL (L/h)	66.7 (57.3–78.5)	44.2 (37.9–50.6)
*V*_1_ (L)	395 (339–446)	647 (394–905)
Q (L/h)		601 (474–707)
*V_p_* (L)		19100 (16,700–21,700)
*K_a_* (1/h)	2.11 (1.22–3.18)	0.108 (0.0797–0.136)
MTT (h)	0.987 (0.72–1.31)	1.23 (0.91–1.723)
NN	7.53 (5.10–13.7)	5.42 (3.56–8.01)
F	1.00 fixed	1.00 fixed
Additive error (ng/mL)	0.20 fixed^*c*^	0.594 (0.345–0.892)
Proportional error (%)	30.7 (26.2–34.7)	25.1 (22.2–27.6)

Interindividual variability (% CV)^*d*^		
CL	18.6 (12.6–24.5)	19.9 (12.0–45.0)
* K_a_* (1/h)	45.7 (3.33–112)	37.0 (24.6–52.2)
MTT (h)	49.2 (32.9–76.2)	80.6 (61.5–108)
F	41.1 (29.7–55.8)	32.7 (25.4–43.4)

a CL, clearance; *V*_1_, volume of distribution in the central compartment; Q, intercompartmental clearance; *V_p_*, peripheral volume of distribution; *K_a_*, absorption rate constant; MTT, absorption mean transit time; NN, number of absorption transit compartments; F, relative bioavailability. All clearances and volumes of distribution refer to a patient weighing 55 kg and a fat-free mass (FFM) of 45 kg. All clearance and volumes of distribution were allometrically scaled using the body weight (wt) expected for naphthoquine, whose clearance was allometrically scaled using fat-free mass. The corresponding parameters scaled to a typical male adult weighing 70 kg (body surface area [BSA], 1.73 m^2^; FFM, 56.1 kg) are as follows: CL = 52.0 L/h, *V*_1_ = 823 L, Q = 744 L/h, and *V_p_* = 24,300 L. CL/F = θ_pop_ × (FFM/45)^0.75^ for naphthoquine, CL/F = θ_pop_ × (wt/55)^0.75^ for artemisinin, and *V*/F = θ_pop_ × (wt/55) for naphthoquine and artemisinin, where θ_pop_ is the population estimate.

b The 95% confidence intervals (CIs) were obtained by the SIR procedure.

c Additive error was fixed to 20% of the LLOQ value.

d Interindividual variability was assumed as log-normally distributed and is reported as approximate %CV calculated as (estimate)^½^ × 100.

**FIG 3:**
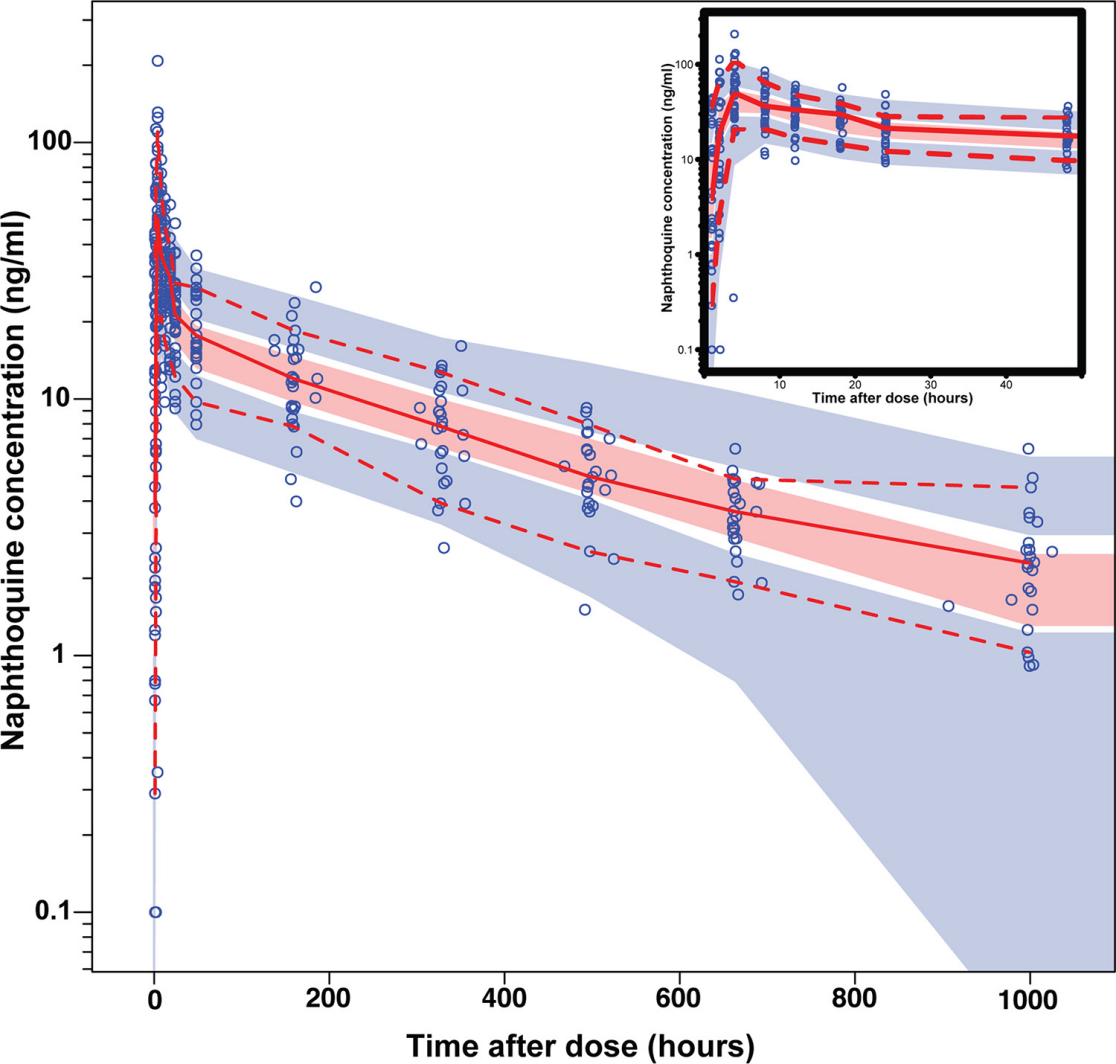
Visual predictive check of the final model describing the plasma concentrations of naphthoquine versus time in uncomplicated malaria patients from Tanzania. Open circles are the observed data points, solid and dashed lines are the 50th, 5th, and 95th percentiles of the observed data, and shaded areas are the simulated (*n* = 1,000) 95% confidence interval for the same percentile.

For naphthoquine, not all patients contributed 13 samples as per protocol (median, 13 samples per patient; range, 9 to 13) and only 363 naphthoquine concentrations were available, of which five (1.4%) were below the limit of quantification (BLOQ). Two samples were not realistic and assigned to the category “missing.” The naphthoquine concentration-time profile was best described by the two-compartment disposition model with a transit compartment absorption phase. The transit compartment model was superior to a lag time model (ΔOFV = −65.7 versus −23.8). For the distribution phase, a three-compartment model did provide a slightly better fit than a two-compartment model (ΔOFV = −11.3, 2 degrees of freedom [df], *P* = 0.0035), this additional complexity made the model parameter estimates unstable and implausible; therefore, a two-compartment model was selected. Figure 1 and Table 2 depict the final structural model and parameter estimates, with sampling importance resampling (SIR) used to estimate the precision on the parameters (*n* = 500). Allometric scaling of the clearance parameter using fat-free mass (FFM) was better than that with body weight (ΔOFV = −14.4 versus −23.2), and body weight was slightly better than fat-free mass for volume parameters (ΔOFV = −10.0 versus −8.9). Adding other available covariates (sex, age, fever, hemoglobin, temperature, and hematocrit) did not improve the model fit. A clearance of 44.2 L/h was estimated for a typical individual with a fat-free mass of 44.3 kg. A visual predictive check indicates the model described the data well (Fig. 3). Basic goodness-of-fit diagnostic plots are presented in Fig. S1B in the supplementary material; these plots showed no overall obvious model misspecification and suggested that the developed model has adequate predictive performance.

### Simulations.

**FIG 4:**
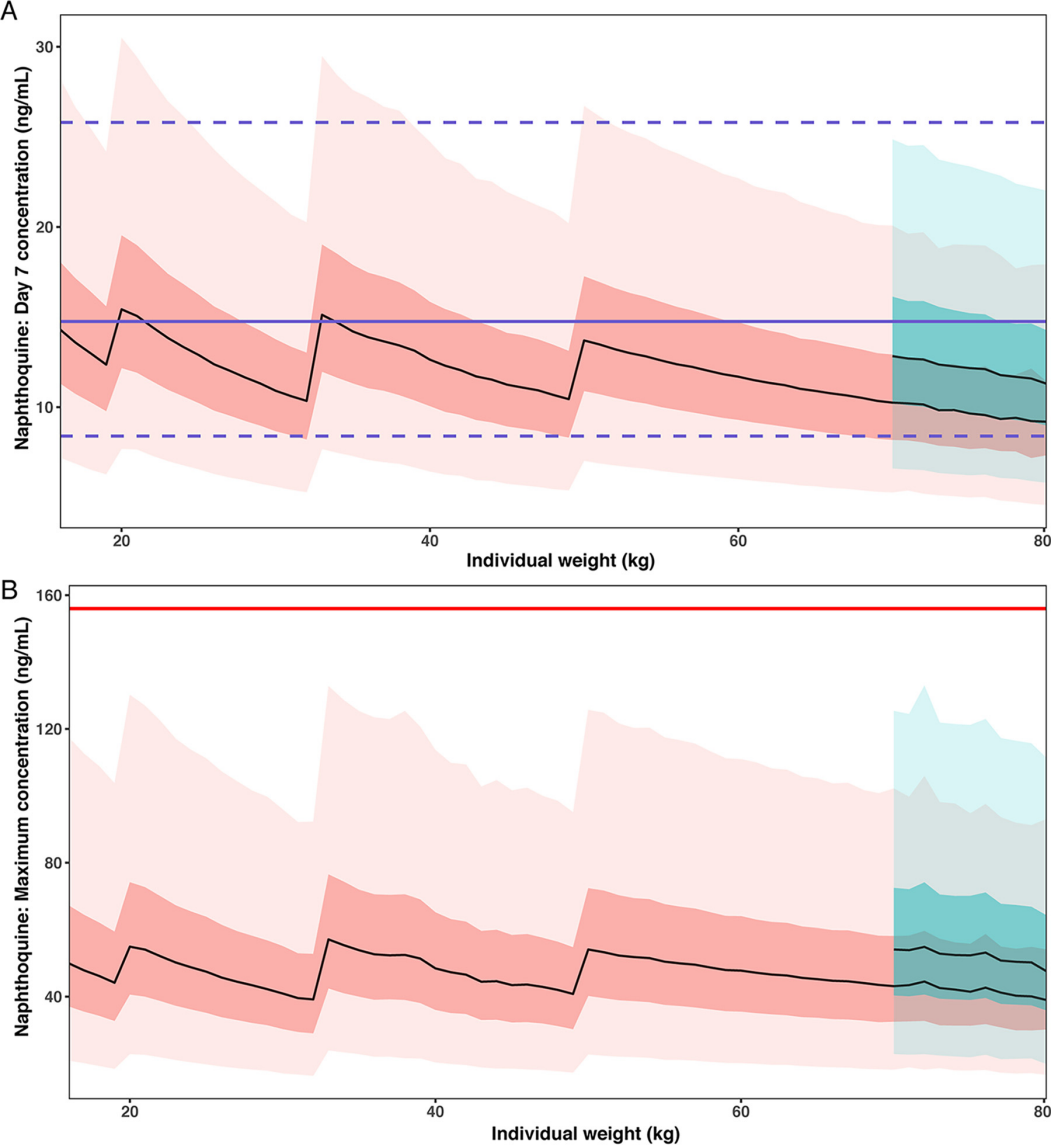
Simulation results of day 7 plasma naphthoquine concentration (A) and maximum concentration of naphthoquine (B). Results from the current recommended dose are in coral, and those from the optimized dose regimen are in blue. The purple line in panel A is the median, the dashed lines are the 5th and 95th percentiles of the simulated efficacy target, respectively, and the red line in panel B represents the target *C*_max_ (156 ng/mL). Simulations of weight are presented as a distribution plot: the median is represented by the black line, the thick shading represents the 25th and 75th percentiles, and the lighter shading represents the 5th and 95th percentiles.

The naphthoquine day 7 concentration of patients weighing 47.8 ± 4.3 kg was predicted to be 14.5 (IQR = 11.7 to 18.6) ng/mL. Figure 4A summarizes the results for different weight ranges using the currently recommended dose and indicates that overall, the simulated day 7 concentration are in line with the defined efficacy target range. However, the day 7 concentration of naphthoquine for individuals who weigh ≥70 kg was lower than the lowest concentration from the previous weight bands. Therefore, new dose and weight bands were explored for individuals who weigh ≥70 kg.

A new dosing regimen for different weight bands using these optimized thresholds is summarized in Table 3, together with the currently recommended doses. The optimized dosing regimen includes higher doses per kg for patients of higher weight to achieve comparable exposure across weight bands without any risk of toxicity for both artemisinin (see Fig. S3 in the supplemental material) and naphthoquine (Fig. 4B).

**TABLE 3:** Current dose regimen and optimized dose regimen based on simulations for naphthoquine^*a*^

Current dose regimen		Simulation-based dose regimen	
Body wt (kg)	ART/NQ (mg)	Body wt (kg)	ART/NQ (mg)
16–20	375/150	16–20	375/150
21–32	500/200	21–32	500/200
33–49	750/300	33–49	750/300
≥50	1,000/400	50–69	1,000/400
		≥70	1,250/500

a ART, artemisinin; NQ, naphthoquine. The group whose dose was optimized is highlighted with shading.

## DISCUSSION

There is increasing interest in developing novel or updated combination therapies, particularly single-dose therapies, as alternatives to current 3-day regimens in sub-Saharan African countries where malaria is endemic, including Tanzania. Previously, one such combination considered was artemisinin and naphthoquine prior to recent and unexpected toxicity findings in animal toxicity studies (15). Several studies have examined the pharmacokinetic properties of artemisinin in populations from areas of malaria endemicity when the drug is given alone or in combination with other partner drugs as a single-dose therapy. However, despite use in routine clinical practice and several PK studies conducted in Papua New Guinea (PNG), this is the first time a population PK analysis of naphthoquine given in combination with artemisinin has been reported in a Tanzanian population. Characterization of the PK of any drug, including naphthoquine is essential to ensure evidence-based optimized dosing and to ensure appropriate regimens from single to multiple dosing are chosen.

Despite extensive evidence of artemisinin PK dynamics, there is still some uncertainty about the most appropriate model for different dosing regimens. In our analysis, we found a one-compartment model best described artemisinin pharmacokinetics, which is similar to several previous studies (37, 38). In contrast, other studies in both healthy volunteers and malaria patients reported first-order elimination with one- or two-compartmental disposition, and a semimechanistic model with first-pass hepatic extraction and autoinduction of clearance (37–42). The differences between these reports are most likely due to the single artemisinin dose and limited PK data in the follow-up of this study. Artemisinin has been reported to induce its own metabolism when repeated doses are given (43), which could explain the better fit of autoinduction models in studies where patients were treated with multiple doses of artemisinin. In the present study, only a single dose was given, and drug concentrations were only available for 18 h after the dosage. Thus, the autoinduction model could not be tested. Moreover, in this study, no covariate relationship other than allometry was found to improve our population pharmacokinetic parameters. Nevertheless, the parameter estimates are in line with the previous population pharmacokinetic study with adult patients after adjusting for body weight (38) and with a noncompartmental analysis (44).

In contrast to artemisinin, naphthoquine has few PK studies. Previous findings from an artemisinin-naphthoquine study in PNG (14), reported that a three-compartment disposition model with transit compartment absorption best described the population pharmacokinetics of naphthoquine. Similar findings have been reported for other quinolone antimalarials: for example, chloroquine (45–47) and piperaquine (36, 48). Nevertheless, we found a two-compartment model more suitable because a three-compartment model was unstable and gave implausible clearance estimates. In the PNG study, patients with fever were associated with a 32% decrease in relative oral bioavailability. Furthermore, the same PNG study found a 1-g/dL increase in patient hemoglobin level was associated with a 16% increase in the volume of distribution of the central compartment (*V*_1_) (14). In the present Tanzanian analysis, no appropriate parameter-covariate relationship was found. In addition, we found the volumes of distributions were lower in our study than in the PNG study (14), even after adjusting for body weight allometric scaling. The higher volume might be attributed to the lower bioavailability due to fever in the PNG study (14).

Despite not being considered as a potential treatment candidate at this time, we undertook a simulation analysis to assess the manufacturer’s dose recommendation compared to our defined PD thresholds. Our simulations suggest that the manufacturer’s current dose recommendation might be too broad, resulting in slight underdosing of patients weighing ≥70 kg (Fig. 4). We thus defined new dosing for these patient groups in order to achieve optimal plasma concentrations (based on the 47-kg adult). Similar findings have been reported previously for the combination of artesunate and amodiaquine (49). For example, individuals in weight bands over 65 kg are likely underdosed as all treatment failures observed in unrelated artemether-lumefantrine studies (3, 4) were in participants over 65 kg. Our preliminary revised dosage scheme for naphthoquine indicates a higher dose in patients with high body weight and will likely result in similar plasma naphthoquine exposure across all weight groups without risk of toxicity (Fig. 4B), even when using tablet strengths in line with currently manufactured tablets.

Given the fixed formulation of ART-NQ, the proposed optimized single-dose results in a median dose of artemisinin 18.8 mg/kg (interquartile range [IQR], 17.2 to 20.8 mg/kg), slightly higher than the recommended 17.2 mg/kg. Artemisinin has previously been administered at higher doses (23.8 mg/kg) together with naphthoquine (9.5 mg/kg) to children 5 to 12 years of age with uncomplicated malaria in PNG (12). Despite the dose being well tolerated, with no serious adverse events in humans, QTc prolongation has been observed in children on artemisinin-naphthoquine 4 h after the third dose in PNG (when given as a 3-day regimen) (50). There have also been reports of central nervous system and hepatic vasculocentric toxicity in beagle dogs (15). Our recommended dose regimen was constructed to ensure that the median day 7 plasma naphthoquine concentration was above 5th percentile of the predicted target. There is a need to evaluate the safety and tolerability of any increased doses, considering the toxicity previously reported (15, 50).

Our simulation analysis included children with body weights of 16 to 19 kg that were not covered in the Tanzanian study. We included lower body weights in our simulations to understand likely exposure in this weight category. However, our simulation results should be interpreted with caution. If the combination ART-NQ was further considered, weights under 16 kg should be informed with data from other trials. Furthermore, studies in infants (<2 years old) must also be completed to estimate the effect of maturation.

Our study has several limitations, primarily concerned with using data from a trial with a small number of patients treated with a single dose of ART-NQ. The study was not powered to detect PK-pharmacodynamic (PD) relationships; thus, we could only assess exposures from the literature to define reasonable PD (instead of defining our PK-PD target from the study). When defining this target, we did not have direct values of concentrations, but we had to use our model to simulate the target exposure. While we believe this was a reasonable approach, we could not account for any factors that may have caused the pharmacokinetics to be different between the target study and ours (e.g., in study population, drug formulation, or administration procedure). Further clinical studies and pooled analysis of all PK studies in multiple populations of naphthoquine alone, or in combination, are warranted—in particular, the inclusion of PK studies in a pooled population PK-PD analysis that includes PD data (e.g., recrudescence).

In conclusion, this study contributes further evidence on single-dose combination therapy previously considered for malaria. We described the population pharmacokinetic properties of artemisinin and naphthoquine in patients with uncomplicated malaria in Tanzania and via model simulations found that larger adults (≥70 kg) would experience lower naphthoquine exposure than lighter adults based on the current dose recommendation of 8 mg/kg.

## MATERIALS AND METHODS

### Study area and design.

Pharmacokinetic (PK) data was obtained from an ART-NQ phase IV, single-center, 2-arm randomized controlled study that evaluated the safety, tolerability, efficacy, and pharmacokinetics of ART-NQ compared to dihydroartemisinin piperaquine phosphate (Eurartesim). The study was conducted in 2014 at the Bagamoyo Clinical Trial Unity (BCTU) in the Bagamoyo District, about 74 km north of Dar es Salaam, within the coastal region of Tanzania. Patients with malaria symptoms residing within the Bagamoyo District seeking care at the health facilities were informed about the study, and those interested were tested for malaria using rapid diagnostic tests (RDTs). Parasite-positive patients who had given verbal consent were transferred to the facility for screening and inclusion in the study. Written informed consent was obtained from each patient prior to any study procedure. For children under 18 years of age, full written consent was provided either by a parent or by a legal representative, in addition, for children between 12 and 17 years of age, the child gave written assent. The study was approved by the Tanzania Food and Drug Authority (TFDA) and by the institutional review boards of Ifakara Health Institute (IHI-IRB) and the National Institute for Medical Research (NIMR) respectively. Patients were hospitalized for 3 days and then discharged and followed up over a period of 42 days.

### Drug regimen and blood sampling.

Patients randomized to ART-NQ received a single dose of standard treatment on day 0. Each tablet of ART-NQ contains 125 mg artemisinin and 50 mg of naphthoquine. The total dose for adults was 1,000 mg of artemisinin and 400 mg of naphthoquine (8 tablets), and for children, the dose was based on body weight (20 mg/kg of body weight for artemisinin and 8 mg/kg for naphthoquine) (Table 3). The drug was orally administered under supervision. The study medication was administered 3 h apart from food.

Blood samples (3 mL) were collected from each patient to obtain measurements of artemisinin and naphthoquine concentrations in plasma. The samples (for both adults and children) were collected 30 min prior to dosing (predosing) and thereafter (postdosing) at 1, 2, 4, 8, 12, and 18 h for both artemisinin and naphthoquine and then on days 4, 7, 14, 21, 28, and 42 for naphthoquine only. Plasma was separated from whole blood into cryovials and stored at BCTU at −80°C before transfer to Swiss BioQuant (Reinach, Switzerland) for analysis.

### Analytical methods.

The quantification of artemisinin and naphthoquine concentrations in plasma was performed by column separation with reverse-phase chromatography followed by detection with triple-stage quadrupole tandem mass spectrometry (MS/MS) in the selected reaction monitoring mode. Three independent quality control samples at different concentrations were analyzed within each batch to ensure accuracy and precision during analysis. For artemisinin, the quality controls were performed with concentrations of 3.0, 50.0, and 375.0 ng/mL, and for naphthoquine, the quality controls were performed at 0.6, 5.0, and 37.5 ng/mL. The coefficients of variation (%CV) during artemisinin quantification (*n* = 16 at each concentration) were 8.5%, 3.5%, and 5.7% at 3.0, 50.0, and 375 ng/mL, respectively, and for naphthoquine (*n* = 12 at each concentration), they were 6.0%, 5.0%, and 4.7% at 0.6, 5.0, and 37.5 ng/mL, respectively. The lower limits of quantification (LLOQ) were set at 1 and 0.2 ng/mL for artemisinin and naphthoquine, respectively.

### Data analysis and pharmacokinetic modeling.

Preparation of data sets for the analysis and calculation of summary statistics on age, weight, sex and other demographics, as well as vital and laboratory parameters, was undertaken using Stata version 13 (Stata, College Station, TX, USA).

The population pharmacokinetics of artemisinin and naphthoquine plasma concentration-time data were analyzed using nonlinear mixed-effects methods in NONMEM version 7.3 (Icon Development Solutions, Ellicott City, MD). The first-order conditional estimation method with interaction (51) was used for estimation of the population parameters.

Models were fitted separately for artemisinin and naphthoquine. Different disposition models (one, two, or three compartments) with first-order elimination and first-order absorption with either lag times or transit compartments (52) were evaluated. The numbers of transit compartments were estimated from the data. Allometric scaling was included to adjust for the difference in body sizes between adults and children, using the suggested exponents of 1 for volumes of distribution and 3/4 for clearance terms. (53). Total body weight, fat, and fat-free mass (FFM) were tested as body size descriptors (54). FFM was derived for males and females separately (55). Drops in the NONMEM objective function value (OFV) and inspection of goodness-of-fit plots and visual predictive checks (VPCs) were used to guide the selection of suitable models (56). The OFV as calculated using NONMEM approximately follows a χ^2^ distribution. Interindividual variability terms were introduced after each step of the structural model development one by one, and those that were not different from zero were removed. The variability terms in each parameter were described using a log-normal distribution. Relative bioavailability (F) was fixed to 1, and interindividual variability in bioavailability was estimated. A combined additive and proportional error model were used to describe residual unexplained variability, and the M6 method suggested by Beal (57) was used to handle values below the limit of quantification. M6 was chosen instead of the M3 and M4 methods because the M6 method is easily implementable, and the statistical loss of using M6 is often not large when compared to M3 and M4. Briefly, values below the limit of quantification were imputed to half the lower limit of quantification, except for trailing values in a consecutive series, which were ignored for the model fit, but included for diagnostic plots.

The relationships between model parameters and the baseline covariates age, parasitemia, hemoglobin, hematocrit, glomerular filtration rate, body mass index, sex, and fever were evaluated using stepwise covariate modeling. Glomerular filtration rate was derived from serum creatinine using the Cockroft-Gault equation for individuals above the age of 16 and the Schwartz equation for those below 16 years (58, 59). To unify the estimates from the two equations, we standardize the set of values derived from the Cockroft-Gault equations to a 70-kg adult (53). A stepwise forward inclusion algorithm (*P* < 0.05) and backward elimination (*P* < 0.01) were used (60). Parameter precision was obtained by the sampling importance resampling (SIR) method (61).

### Simulations.

Stochastic simulations of the final model were performed to explore exposures achieved with the current dosing regimen and to possibly optimize it. The day 7 concentration was used as the exposure that best relates to efficacy (62), while the maximum concentration of drug in serum (*C*_max_) was monitored as a measure of safety.

To define the efficacy target range for day 7 concentrations, we used a study by Tun et al. (11) reporting that 400 mg of naphthoquine in combination with artemisinin had a cure rate of 98%. Tun et al. did not report pharmacokinetic results, so we used our model to predict the expected concentrations given the dose and body weight of the patients in the study (400 mg of naphthoquine given to patients weighing 47.8 ± 4.3 kg), hence establishing an efficacy target range. Then we assessed whether our simulated day 7 concentrations are in line with the simulated efficacy target range and in harmony with the concentrations from the other weight bands.

To define the cutoff for safety, we referred to a study by Wang et al. (21), who administered a single dose of 600 mg naphthoquine phosphate to 14 healthy volunteers and concluded that the dose was safe. The range of *C*_max_ values was from 98.9 to 245.2 ng/mL, so we decided to use the geometric mean value of 156 ng/mL for our safety evaluation.

For the simulations to be relevant for a population of malaria patients, we used individual demographic data from malaria patients from Burkina Faso, Ghana, Mozambique, and Tanzania (*n* = 833), obtained from the INDEPTH network-INESS study (63), and from a malaria surveillance study (*n* = 500) from the Bagamoyo Research and Training Center (Ifakara Health Institute, Tanzania) (data not published). A total of 1,333 *in silico* patients were thus available for simulation with a minimum weight of 16 kg, the minimum recommended by the drug manufacturer. We performed 5,000 simulations of the entire *in silico* population to evaluate the day 7 concentration and *C*_max_, and subsequently, 3,000 values in each 1-kg weight band (1-kg interval) were randomly drawn from the simulation results. We first used the dosing regimen recommended by the manufacturer (Kunming Pharmaceuticals, Kunming, China) shown in Table 3, then we attempted to optimize it.
