# Supplementary material for: Population Pharmacokinetics of Antimalarial Naphthoquine in Combination with Artemisinin in Tanzanian Children and Adults: Dose Optimization
Source: Antimicrob Agents Chemother. 2022 Apr 25;66(5):e01696-21. doi: 10.1128/aac.01696-21 (PMC9112936; doi:10.1128/aac.01696-21)
Supplement: Supplemental file 1 — Fig. SA1 to SA3. Download aac.01696-21-s0001.pdf, PDF file, 0.5 MB [file aac.01696-21-s0001.pdf]

**Supplemental data**

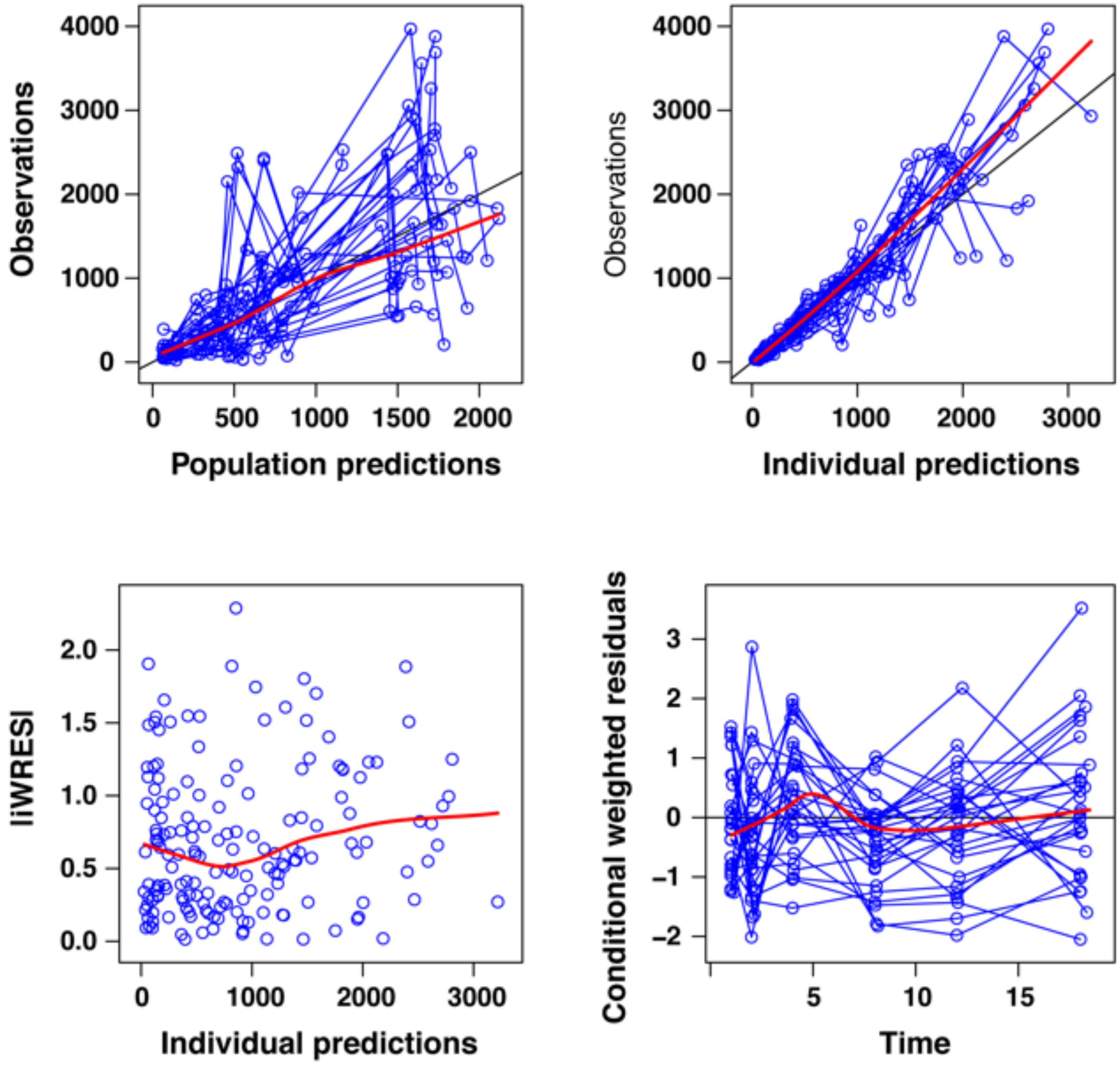


Figure A1A. Basic goodness-of-fit diagnostic of the final population pharmacokinetic model of artemisinin in uncomplicated P. falciparum malaria patients. Open blue circles are the observed data points; red lines, locally weighted least-squares regression; black lines, line of identity.

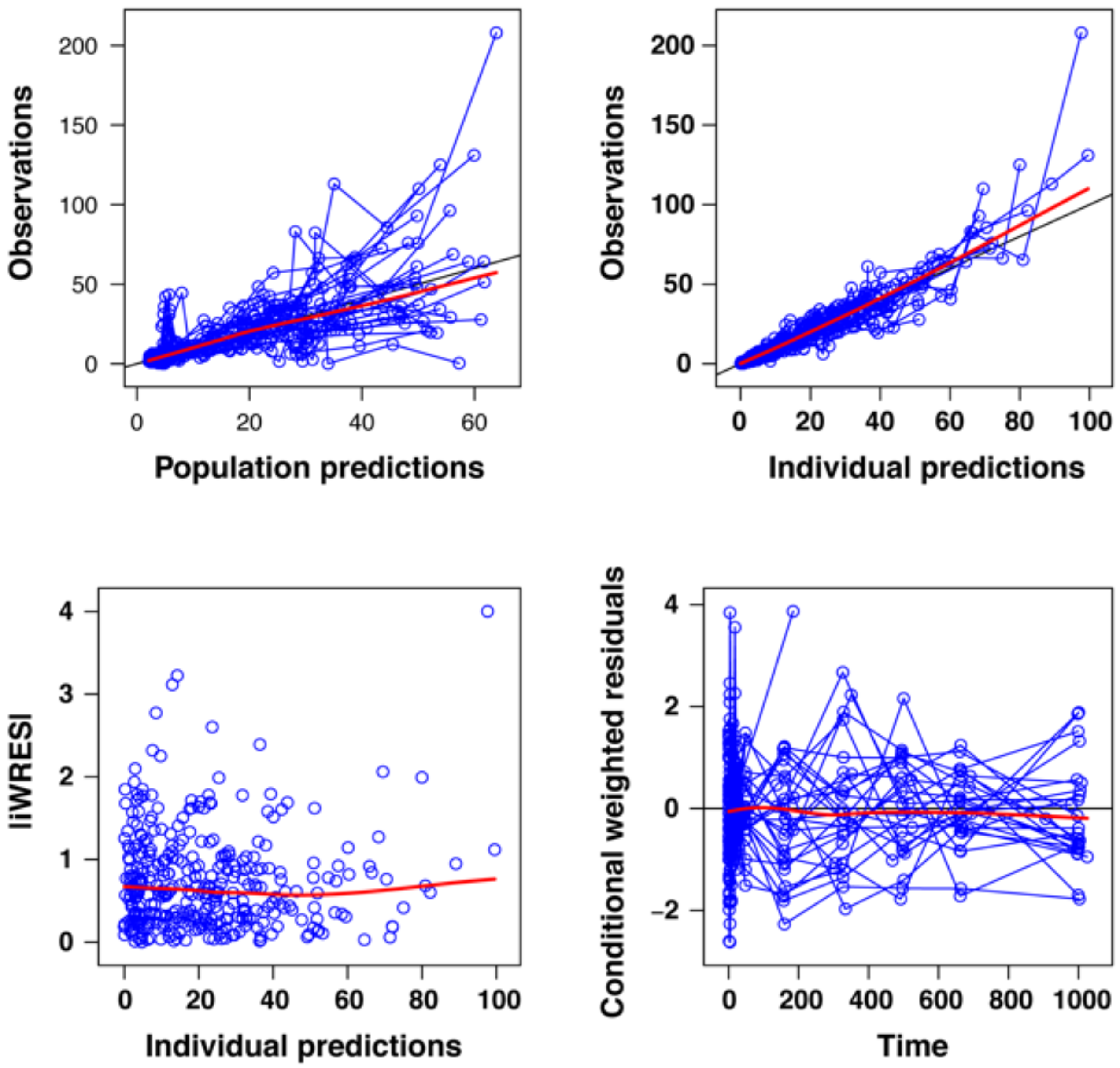


Figure A1B. Basic goodness-of-fit diagnostic of the final population pharmacokinetic model of naphthoquine in uncomplicated P. falciparum malaria patients. Open blue circles are the observed data points; red lines, locally weighted least-squares regression; black lines, line of identity.

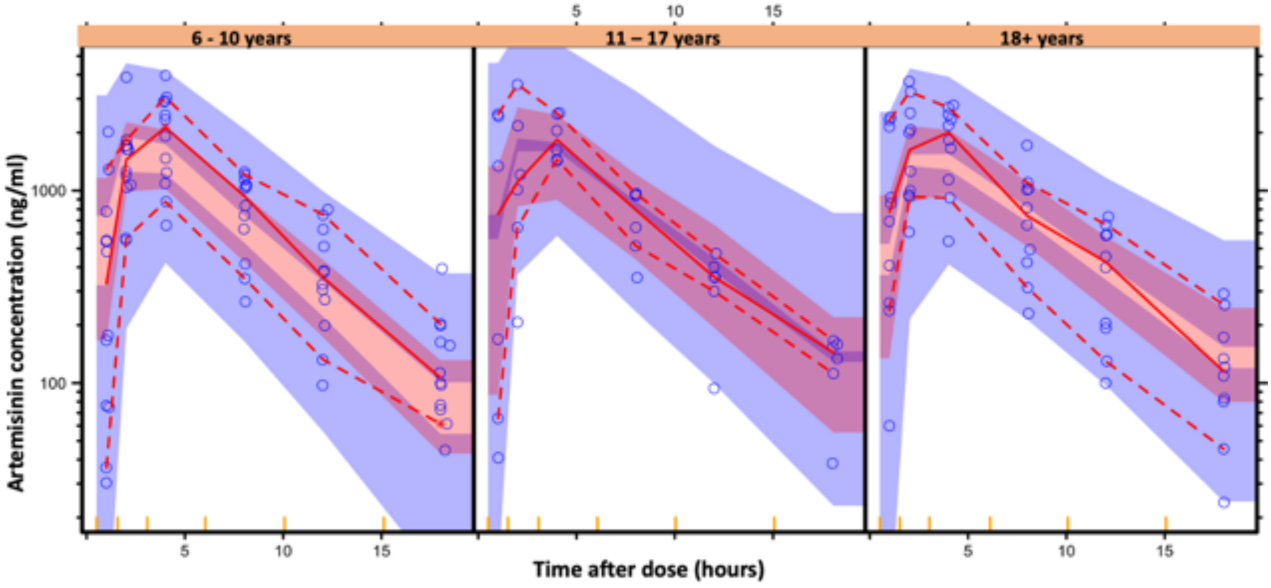


Figure A2A. Visual predictive check of the artemisinin final model stratified by age group. Open circles are the observed data points; solid and dashed lines are the 50th, 5th, and 95th percentiles of the observed data; shaded areas are the simulated (n=1000) 95% confidence interval for the same percentile.

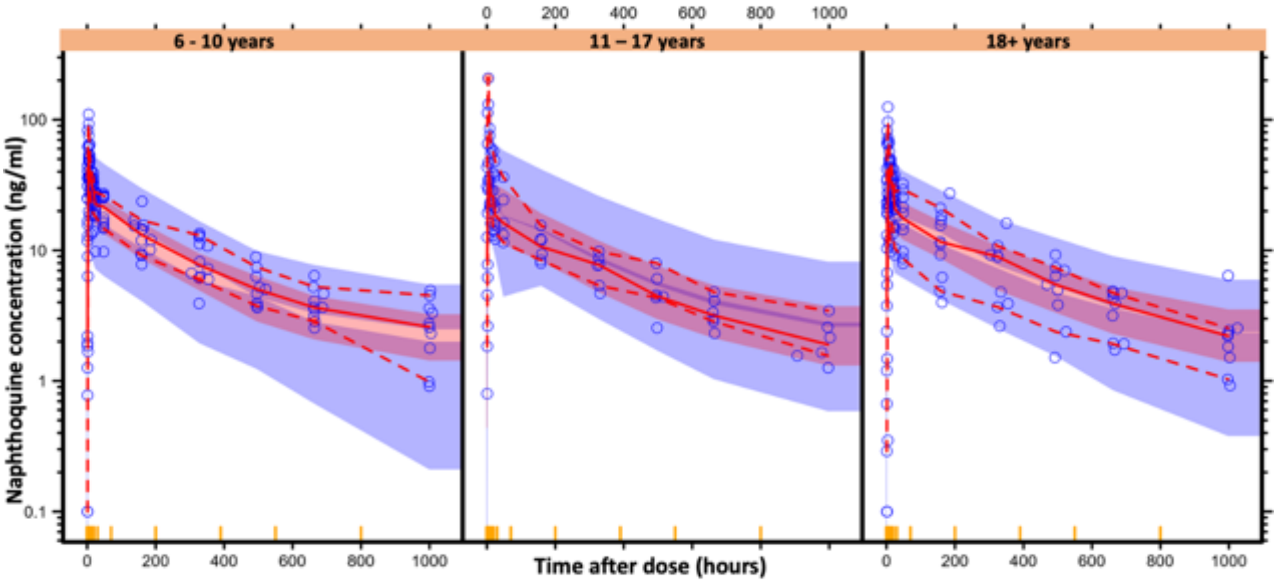


Figure A2B. Visual predictive check of the naphthoquine final model stratified by age group. Open circles are the observed data points; solid and dashed lines are the 50th, 5th, and 95th percentiles of

the observed data; shaded areas are the simulated (n=1000) 95% confidence interval for the same percentile.

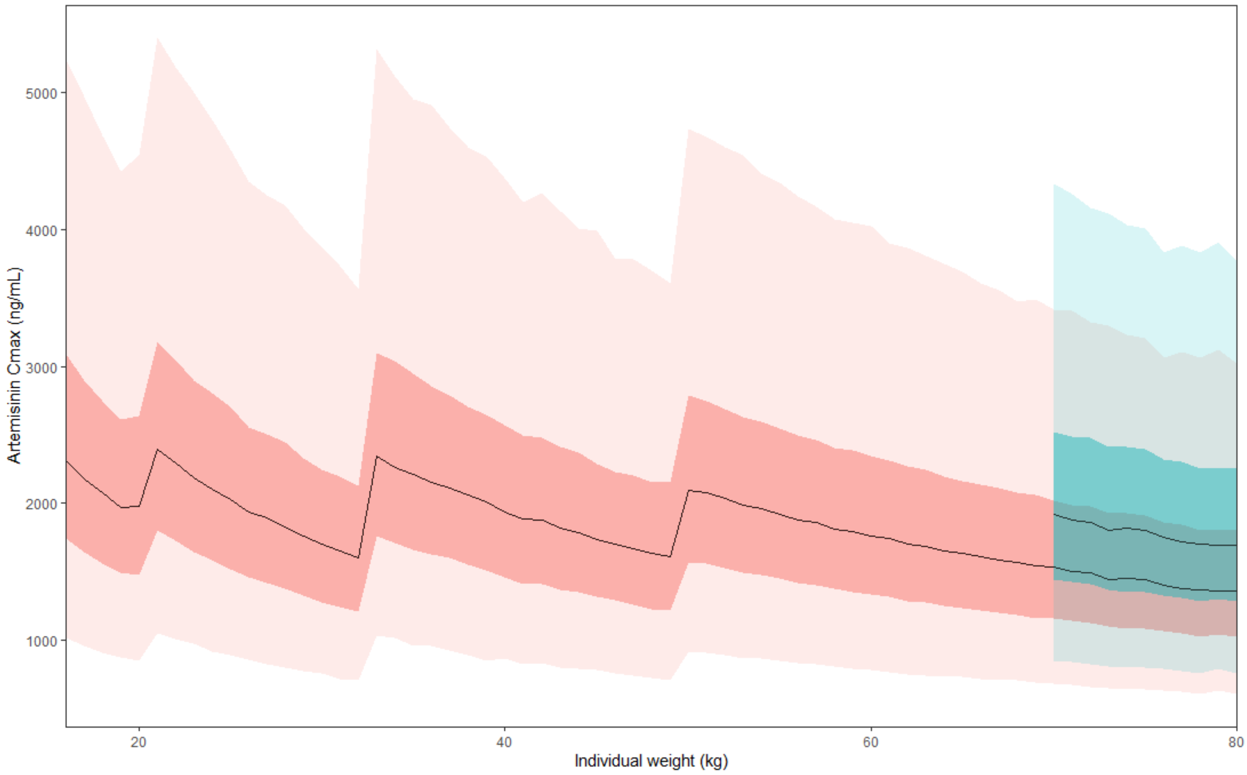


Figure A3: Simulation of maximum concentration of artemisinin across the weights. Results from the current recommended dose are in coral and optimized dose regimen in blue. Simulations of weight are presented as distribution plot, the median is the black line, the thick shading is 25th and 75th percentile, and the lighter shading is the 5th and 95th percentiles.
